# Complete chloroplast genome of *Helicia nilagirica* Bedd. and its phylogenetic analysis

**DOI:** 10.1080/23802359.2019.1703587

**Published:** 2019-12-18

**Authors:** Ying-Feng Niu, Jin Liu

**Affiliations:** Yunnan Institute of Tropical Crops, Xishuangbanna, China

**Keywords:** *Helicia nilagirica* Bedd., chloroplast genome, phylogenetic analysis

## Abstract

*Helicia nilagirica* Bedd. is a medicinal plant. Its fruit is the main raw material from which helicid, a compound that has sedative, hypnotic, analgesic, and other central nervous system inhibitory effects, is extracted. Here, we report and characterize the complete chloroplast genome of *H. nilagirica* in this study. The complete chloroplast genome of *H. nilagirica* contains 157,217 bp and consists of four regions, including a large single-copy region (LSC, 85,516 bp), two inverted repeat regions (IRs, 26,700 bp), and a small single copy (SSC, 18,301 bp) region. A total of 132 genes were obtained by annotation, including 88 protein-coding genes, 36 transfer RNA (tRNA) genes, and 8 ribosomal RNA (rRNA) genes. Phylogenetic analysis showed that *H. nilagirica* is closely related to *Macadamia integrifolia*, suggesting that *H. nilagirica* may be used as rootstock or gene donor in macadamia breeding. This study lays a foundation for future phylogenetic and evolutionary studies of Proteaceae.

*Helicia nilagirica* Bedd. is a medicinal plant (Wu et al. 2004), distributed throughout China, India, Myanmar, and other Southeast Asian countries. Its fruit is the main source for helicid, which has sedative, hypnotic, analgesic and other central nervous system inhibitory effects (Chen et al. 2008). In addition to helicid, the fruits and leaves of *H. nilagirica* also contain a variety of other compounds (Wu et al. 2010), including p-hydroxybenzaldehyde, p-hydroxybenzoic acid, gallic acid, and others (Liu et al. 2005a, 2005b). They have been used for centuries to treat diseases among the ethnic minorities found in Yunnan, China. Although the wild germplasm resources of *H. nilagirica* have declined sharply in recent years, there is still only a small amount of research on its geographic distribution, dispersal, and evolutionary relationships.

Chloroplast genome is a powerful tool by which the evolutionary relationships of species can be studied (Du et al. 2017; Liu et al. 2018). This is due to two characteristics: (1) the chloroplast genome contains abundant genetic information and (2) it is relatively highly-conserved in evolution. Although it is an important tropical plant and medicinal plant, the mitochondrial and chloroplast genomes of *H. nilagirica* have not yet been reported in the literature.

Here, we report and characterize the complete chloroplast genome of *H. nilagirica*. Young leaves were collected from the rooted plants of *H. nilagirica* at the Germplasm Resource Nursery of Yunnan Institute of Tropical Crops (coordinates are N 22°00′56.69″ and E 100°46′48.10″) for genomic DNA isolation using the Dneasy Plant Mini Kit (Qiagen). Isolated DNA was stored in the ultra-low temperature specimen library of YITC (specimen accession number: YITC-2019-FZ-P-017). A Roche/454 system (Roche 454 Life Sciences) was used for DNA sequencing and CLC Genomic Workbench v3.6 (http://www.clcbio.com) was used for genome assembly, while DOGMA (Wyman et al. 2004) was used to assemble and annotate the chloroplast genome. Corrections were made manually when required. The results were submitted to GenBank under accession number MN714870.

The complete chloroplast genome of *H. nilagirica* contains 157,217 bp and consists of four regions, including a large single-copy region (LSC, 85,516 bp), two inverted repeat regions (IRs, 26,700 bp), and a small single copy (SSC, 18,301 bp) region. The percentage of DNA bases is 30.62% A, 31.29% T, 18.66% G, and 19.43% C, with a GC content of 38.10%. A total of 132 genes were annotated, including 88 protein-coding genes, 36 transfer RNA (tRNA) genes, and 8 ribosomal RNA (rRNA) genes. The protein coding genes are involved in the following biological functions: photosystem I, photosystem II, cytochrome b/f complex, ATP synthase, NADH dehydrogenase, RubisCO large subunit, RNA polymerase, Ribosomal proteins (SSU/LSU), clpP, matK, hypothetical chloroplast reading frames (ycf), and more.

We constructed a phylogenetic tree of *H. nilagirica* and 16 other species based on their complete chloroplast sequences. *Amborella trichopoda*, which belong to the order Amborellales, was used as the outgroup (Figure 1). MAFFT (Katoh and Standley 2013) was used for multiple sequence alignment and MEGA7.0 (Kumar et al. 2016) was used for maximum-likelihood (ML) analysis. The results show that *H. nilagirica* is closely related to *Macadamia integrifolia*, suggesting that *H. nilagirica* may be used as rootstock or gene donor in macadamia breeding. This study lays a foundation for future phylogenetic and evolutionary study of Proteaceae.

**Figure 1.**
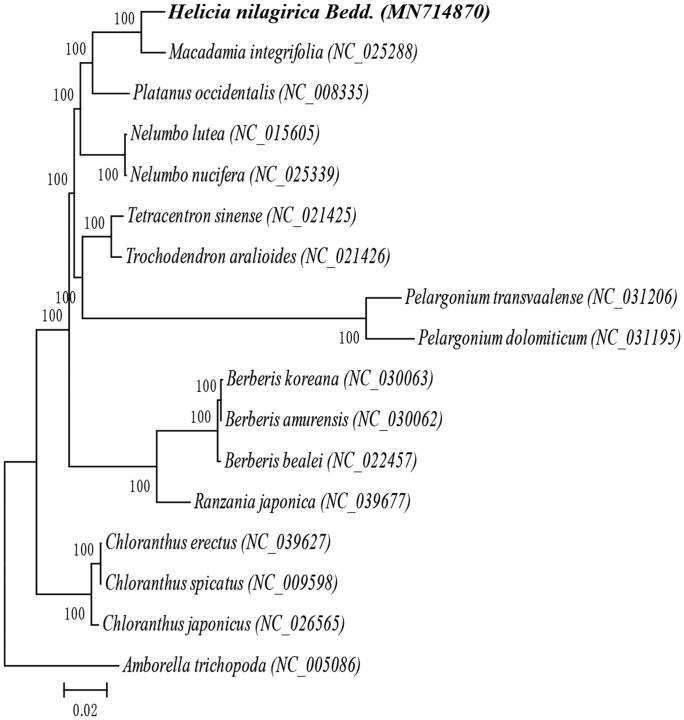
Maximum likelihood phylogenetic tree of 17 species based on complete chloroplast sequences, including *Helicia nilagirica* Bedd. and 16 other species, *Amborella trichopoda* was used as the outgroup. Numbers in the nodes are bootstrap values from 1000 replicates, bootstrap values are shown above the nodes. The species and chloroplast genome accession number in the phylogenetic tree as shown below: *Helicia nilagirica* Bedd. (MN714870), *Macadamia integrifolia* (NC_025288), *Platanus occidentalis* (NC_008335), *Nelumbo lutea* (NC_015605), *Nelumbo nucifera* (NC_025339), *Tetracentron sinense* (NC_021425), *Trochodendron aralioides* (NC_021426), *Pelargonium dolomiticum* (NC_031195), *Pelargonium transvaalense* (NC_031206), *Berberis koreana* (NC_030063), *Berberis amurensis* (NC_030062), *Berberis bealei* (NC_022457), *Ranzania japonica* (NC_039677), *Chloranthus erectus* (NC_039627), *Chloranthus japonicus* (NC_026565), *Chloranthus spicatus* (NC_009598), and *Amborella trichopoda* (NC_005086).
